# Medical Students' Perceptions of the Best Clinical Teaching

**DOI:** 10.1111/tct.70119

**Published:** 2025-06-13

**Authors:** Verneri Hannula, Lari Lehtovirta, Hermanni Liu, Markku Sumanen

**Affiliations:** ^1^ The Wellbeing Services County of Pirkanmaa Tampere Finland; ^2^ Faculty of Medicine and Health Technology Tampere University Tampere Finland

**Keywords:** clinical learning environment, clinical teacher, clinical teaching, medical students, undergraduate medical education

## Abstract

**Introduction:**

Clinical‐phase learning is integral to medical education, increasingly challenged by a rising student population. Prior research has presented conflicting findings on students' preferences for following doctors at different stages of their career. Moreover, evaluations of clerkships across specialties vary significantly. This study explored medical students' perceptions about who they received the best clinical teaching from and why.

**Methods:**

A survey was conducted among 4th‐ to 6th‐year medical students at Tampere University, Finland. Information was sought from participants on aspects regarding from whom and where they believed they received the best clinical teaching: doctors' properties (age group and career stage) and setting (field of specialty and location). Open‐ended responses were analysed using inductive content analysis.

**Results:**

We obtained 262 responses, yielding an overall response rate of 62%. Predominantly, respondents favoured resident doctors (54%), young doctors aged 25–34 years (41%), university hospitals (34%) and the field of internal medicine (25%). Common reasons cited included instructors dedicating time for teaching, enthusiasm about teaching and attentiveness to students. However, over half of the themes were related to specific answer options.

**Conclusions:**

Our findings indicate that Finnish medical students most commonly prefer to receive clinical teaching from residents and young doctors. Nevertheless, exposure to doctors at their different career stages and in various settings is perceived as valuable. To enhance clinical teaching, organisations should allocate adequate time and encourage doctors to engage actively with students, understanding their individual learning needs and fostering independent participation.

## 1 Introduction

Clinical‐phase learning and teaching play a major role in medical education. A significant portion of students' learning occurs while shadowing clinicians and during clerkships. However, teaching in the clinical environment is often not organised optimally [1]. As in many other countries, the number of medical students in Finland has increased [2, 3]. This growth in educational volume places significant demands on students' clinical teaching. In Finland, all faculties conduct part of the clinical teaching outside university hospitals, which has been recognised as a strength in national evaluations [2]. Similarly, decentralisation is often perceived as being advantageous for students [4]. However, this decentralisation also means that doctors frequently engage in clinical teaching without having had formal training in education.

In an earlier Finnish survey, approximately half of the doctors responding reported that they provided clinical teaching, yet only one‐tenth had received any training in teaching methods [5]. Previous studies have produced inconsistent findings regarding from whom and where students prefer to receive their clinical training. It is established that the career stage that doctors are in influences student learning [6, 7, 8] and students' evaluations of their clinical teachers have been found to correlate with their examination scores [8]. However, studies have yielded inconsistent findings regarding whether medical students prefer to receive clinical training from junior or consultant/senior doctors [9, 10]. Additionally, evaluations of clerkships across different specialties vary, with students emphasising a range of aspects when evaluating specialties in hospital and community medicine [11]. Outpatient clerkships are generally rated higher by students compared to clerkships in inpatient clinics [12, 13].

As educational volumes increase, a growing proportion of clinical learning occurs outside formal teaching sessions, conducted by doctors who are not clinical educators. Most previous studies have focused on structured teaching in clinical settings, and the results of these studies have been inconsistent. Therefore, in our study, we investigated students' perceptions of clinical teaching outside structured learning events. We believe that identifying the characteristics of effective clinical teachers and the environments in which quality teaching occurs can help in applying these characteristics more broadly, thereby enhancing the quality of teaching and learning in clinical settings and placements.

*As educational volumes increase, a growing proportion of clinical learning occurs outside formal teaching sessions, conducted by doctors who are not clinical educators*.

Our research question was as follows:

Where and from which types of doctors did students perceive they had received the best clinical teaching and why?

## 2 Methods

### 2.1 Study Context

In Finland, medical school lasts 6 years. At Tampere University, the clinical phase starts after 3.5 years of preclinical studies. During the clinical phase, students follow doctors in both hospitals and health centres (primary care), primarily at Tampere University Hospital. However, the curriculum also includes clerkships at central hospitals and health centres.

At Tampere University, the degree programme also includes 4 months of mandatory internships outside the academic semesters. During the clinical phase and as junior house officers during internships, medical students regularly learn with doctors in consultations, wards, and surgeries [2]. During the spring semester of the fourth year, half of the class participates in 9‐week courses in internal medicine and surgery. The other half of the class completes 6‐week courses in gynaecology, paediatrics and psychiatry during the same semester. In the autumn semester of the fifth year, students take the courses they did not complete in the previous spring. During the spring semester of the fifth year and the autumn semester of the sixth year, students complete clinical courses in the remaining specialties.

In Finland, there are five university hospitals that offer treatment across all specialties. Additionally, there are 16 central hospitals and several smaller regional hospitals. Fifty specialties are recognised in Finland, and medical graduates can pursue specialisation in these fields. Specialist training typically takes 5 or 6 years.

### 2.2 Survey

We gathered perceptions from a broad group of students and integrated both qualitative and quantitative data. Therefore, a survey was selected as the research method, rather than methods such as focus group interviews. Validated questionnaires addressing clinical teaching generally focus on evaluating the learning environment of a single course and do not include open‐ended questions [14, 15]. Therefore, we developed a new questionnaire. The questions were selected by authors' consensus to address the research questions.

An online questionnaire was constructed using Research Electronic Data Capture (REDCap). We conducted a pilot survey with students who had graduated from Tampere University in spring 2020. In the pilot phase, 19 graduates responded to the closed questions of the survey twice, with a two‐week interval between their responses. They also provided feedback on the survey structure. Based on their comments, the categorisation of specialties was modified from five to seven groups, and examples of these groups and different training places were provided.

The survey was distributed to all 4th‐ to 6th‐year medical school students at Tampere University. Participants received personalised survey links via email. Before distributing the links, the study and survey were described during mandatory lectures to all classes. Responses were collected from November to December 2020. In autumn 2021, we published an article based on the second part of the survey in the Finnish Medical Journal [16].

The survey (see Data S1) comprised 10 closed and four open‐ended questions. Participants were instructed to respond according to clinical learning situations in which they received clinical teaching from doctors, excluding formal teaching situations and teaching from designated clinical teachers.

As background information, participants were asked to provide their age, gender and year of study. They were also asked to indicate the locations where they had completed internships as junior house officers, and the specialties involved. Specialties were categorised into seven groups using the classification established by the Finnish Medical Association (see Data S2), with a link provided to this classification. Additionally, participants were queried about the total duration of their internships.

We directed our special interest to the following four questions:

- Which career‐stage doctors do you feel you received the best clinical teaching from?
- What was the age group of those from whom you received the best clinical teaching?
- Which field of doctors do you feel you received the best clinical teaching from?
- Where do you feel you received the best clinical teaching? (university hospital, central hospital, other hospital, health centre)

**FIGURE 1:**
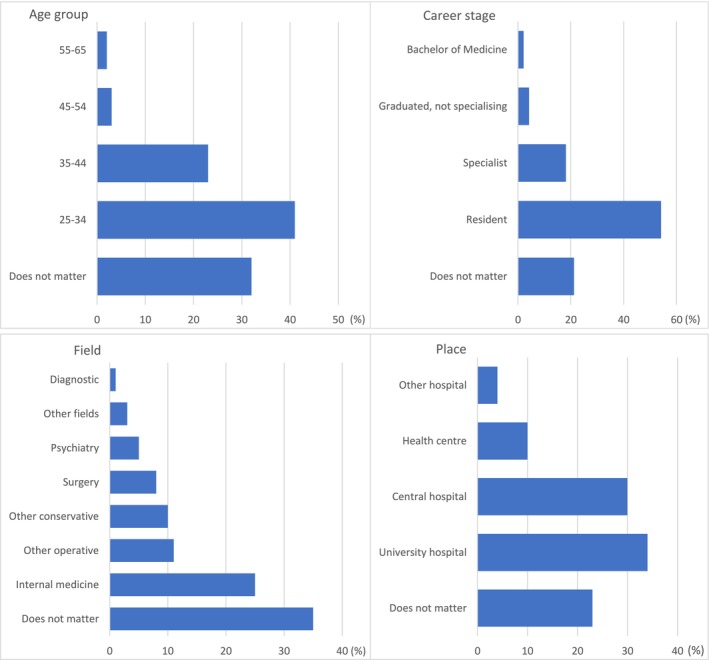
Respondents' answers to the questions on from who and where they had received the best clinical teaching. Answers are presented in percentages. There were 262 answers to questions regarding age and career stage, 261 answers to the question about the field and 260 to the question about the place.

Figure 1 displays all responses for each closed question. Following each closed question, respondents were asked to provide the reasons for their choice.

### 2.3 Ethics

The study received permission from the faculty's Degree Planning Committee. Participation in the survey was voluntary, and participants provided informed consent. The survey did not collect any direct personal information, and the responses could not be linked to the respondents' email addresses. Therefore, ethical committee approval was not required.

### 2.4 Data Analyses

Statistical analyses were conducted using SPSS version 27. Descriptive statistics were computed to summarise the distribution of participants' background information and their perceptions. We examined the influence of background variables (gender, year of study) on responses to the best clinical teaching questions through cross‐tabulation. As the variables were categorical, the chi‐square test was employed; Fisher's exact test was used when more than 20% of the cells had expected counts below 5. A *p*‐value less than 0.05 was considered to be statistically significant.

Open‐ended questions were analysed using inductive content analysis. Responses were read and categorised into themes and categories based on emerging patterns and content [17].

To assess the reliability of our questionnaire, all pilot respondents answered the questions twice, and we calculated kappa values for the four questions related to the best clinical teaching. Kappa values above 0.4 were considered indicative of moderate agreement [18]. The kappa values for each question were as follows: career stage 0.77, field of specialty 0.61, place 0.50. These questions all had *p*‐values less than 0.001, indicating statistical significance. However, the question regarding the age group had a kappa value of 0.38 (*p* = 0.014), which suggests slightly lower agreement compared to the other questions.

## 3 Results

Of the 423 students who received the survey (54% women), 262 responded, resulting in an overall response rate of 62%. Among the respondents, 151 were women, comprising 58% of the total. The median age of the respondents was 25 years, with an age range of 21–42 years. In terms of academic standing, 37% (98) of the respondents were in their 4th year, 35% (92) were in their 5th year and 28% (72) were in their 6th year.

**FIGURE 2:**
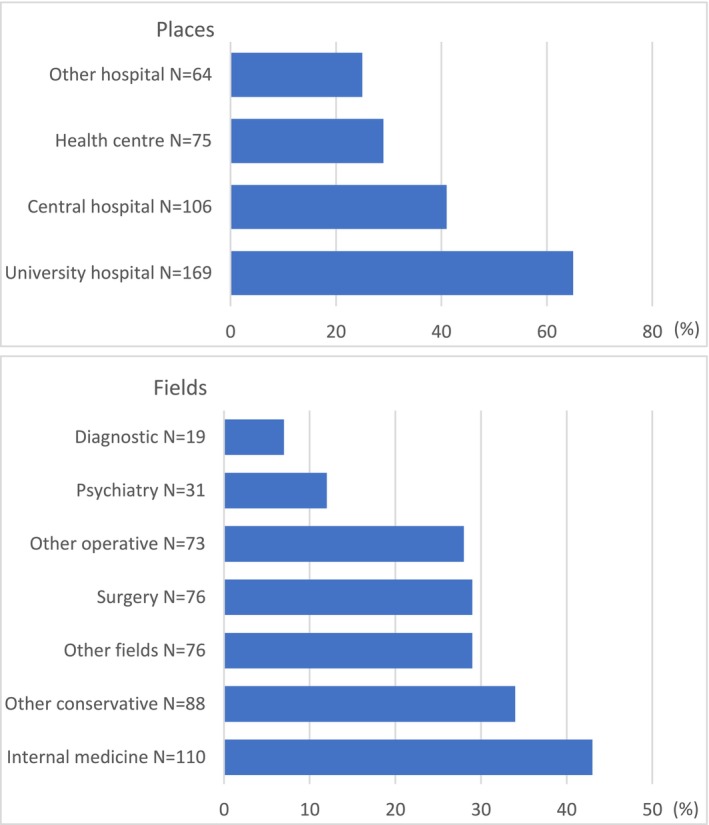
Respondents' internship experience. Answers are presented as percentages. The total number of respondents to both questions was 259, and respondents were able to choose multiple options.

The internship experience of the respondents is illustrated in Figure 2. The mean length of the total internship experience was 2.3 months (median 2.0 months, range 0–10 months).

Evaluations regarding the best clinical teaching are presented in Figure 1. The results, categorised according to the questions, show that the most frequently selected option was resident doctors, accounting for over half of the evaluations. In the age category, young doctors (25–34 years) received approximately two‐fifths of the evaluations. Similarly, university hospitals received around one‐third of the evaluations, while internal medicine fields received about a quarter. The proportion of respondents who indicated that quality ‘does not matter’ varied from one‐fifth (place) to one‐third (field of specialty).

No significant differences were found between genders in responses about the best clinical teaching. However, a significant difference was observed in responses to the question regarding the field of specialty based on the respondents' year of study (*p* = 0.046, Fisher's exact test, see Table 1). There were no significant differences between year groups regarding the other three questions about clinical teaching.

**TABLE 1:** Number of answers to the question ‘From which field of doctors do you feel you have received the best clinical teaching?’ by year of study.

	4th year	5th year	6th year	Total
Does not matter	27	34	31	92
	28%	37%	**43%**	35%
Internal medicine	19	31	16	66
	20%	**34%**	22%	25%
Other operative	15	7	7	29
	**16%**	8%	10%	11%
Other conservative	9	9	8	26
	9%	10%	11%	10%
Surgery	11	7	4	22
	**11%**	8%	6%	8%
Psychiatry	6	4	4	14
	6%	4%	6%	5%
Other fields	8	0	1	9
	**8%**	0%	1%	3%
Diagnostic	2	0	1	3
	2%	0%	1%	1%
Total	97	92	72	261
	100%	100%	100%	100%

*Note: N* = 261. Fisher's exact test revealed a significant difference based on the year of study (*p* = 0.046). In pairwise comparisons, a significant difference was observed between the fourth‐ and fifth‐year students (*p* = 0.09, Fisher's exact test). No statistically significant differences were found between the fourth‐ and sixth‐year students or between the fifth‐ and sixth‐year students. Responses that were more common in one year group compared to the others have been highlighted in green.

Most respondents reported that they had received the best clinical teaching from the place they had completed their internship. Only six out of 96 fourth‐year respondents indicated that they had received the best clinical teaching at a location they had not been an intern at. For fifth‐year students, this number was 21 out of 91, and for sixth‐year students, it was 19 out of 72.

*Most respondents reported that they had received the best clinical teaching from the place they had completed their internship*.

Similarly, most respondents stated that they had received the best clinical teaching in the field where they had internship experience. Only five out of 97 fourth‐year students selected a field in which they had no internship experience. For fifth‐year students, this number was 15 out of 92, and for sixth‐year students, it was 11 out of 72. Some open‐ended responses indicated that during longer internships, the quality of clinical teaching improves as students become more familiar with their instructors.

The themes that emerged from the students' open‐ended responses are presented in Table 2. Some arguments were not specific to any single answer option. These themes included ‘instructors have time for teaching’, ‘instructors are interested in teaching’ and ‘instructors pay attention to students’. However, over half of the themes were related to specific answer options. No specific themes were unique to any particular field of specialty. However, an empathetic approach was notably emphasised in the field of psychiatry. Doctors in internal medicine were perceived as being willing to teach, involving students in working and discussions. Students also noted that topics in internal medicine are broadly useful for all doctors.

**TABLE 2:** The themes and subcategories emerged from the students' open answers.

	Themes	Subcategories	Highlighted in	Quotes
Common to all	Time for teaching	Quality of teaching is dependent on time available. A good instructor has time to teach.		‘The workload is not so hectic, so there is time left to instruct the student.’ (Psychiatry)
	Interest in teaching	Willingness to teach Answering students' questions		‘The person in question was very interested in teaching and wanted to teach alongside the work.’ (Internal medicine)
	Paying attention to students	Informing students about the subject Involving students in the discussion Letting students participate independently Kindness Topics covered are important to students.		‘At the central hospital, I was actively guided and allowed to do things by myself …’
Age and career stage	Excellent skills	Certainty of practices Excellent knowledge of substance	Specialists, older doctors	‘It is probably easier to guide and teach when you have a strong command of your scope of work.’
	Own study times fresh in memory	Understands students' knowledge level Teaches important basics Easily approachable More discussion together	Residents and other young doctors	‘Maybe young people still relate to the student and understand the level of competence better. It is also easy to get along with people of the same age.’
	The advantage of short work experience	Recent information on your own speciality Also show uncertainty Show different consultation practices	Residents and other young doctors	‘… One can see how others cope with their own uncertainties and gets a more realistic picture of the early stages of their careers, e.g. how much young doctors are actually consulting …’
Place	Used to giving teaching	Doctors have learned to teach. Teaching is considered as a duty.	University hospitals	‘University hospitals have generally placed the greatest emphasis on doctors for the teaching of students.’
	Not tired of students	There are less students, so doctors manage to give better teaching. Instructors are easier to get to know in smaller places.	Other places than university hospitals	‘It seems that the presence of students in the central hospital is less frequent, so there is more motivation to teach.’

*Note:* The total count of responses from the open answers across all categories is 555: 191 responses related to the career stage, 107 to age, 132 to fields of specialty and 125 to place.

Students who selected the option ‘does not matter’ often argued that the quality of clinical teaching varies significantly over time and is strongly influenced by the personality of the instructor. Additionally, students reported experiencing both good and poor clinical teaching across a wide range of places and instructors. There were also comments suggesting that a student's own engagement influences the quality of clinical teaching.

## 4 Discussion

In our study, we surveyed medical students' perceptions of where and from which types of doctors they had received the best clinical teaching. Over half of the respondents indicated that they had received the best teaching from residents. In other question categories, the most frequently selected responses included young doctors (aged 25–34 years), internal medicine and university hospitals. Notably, most students reported that the best clinical teaching had occurred at the site where they had completed their internship. Respondents' gender had no significant effect on the responses. However, the year of study had a minor influence on evaluations related to the field of specialty and no significant effect on the other categories. According to the open‐ended responses, the doctors who provided the best clinical teaching were described as having time for students, being kind and willing to teach and demonstrating an understanding of the students' skill levels.

*Over half of the respondents indicated that they had received the best teaching from residents*.

Our finding that most of the medical students reported receiving the best clinical teaching from resident doctors is partly consistent with a study conducted by Gray et al. [9]. In their study, 70% of the respondents felt more comfortable with junior doctors than consultants during bedside teaching. More students preferred bedside teaching from junior doctors compared to consultants (35% vs. 24%). However, a slightly larger proportion trusted the information provided by consultants more than that taught by junior doctors (22% vs. 15%). Similar to our study, Gray et al. also reported that many respondents had no preference between these two groups [9].

In contrast, a study by Alweshahi et al. found that students considered it more important for the bedside teacher to be a senior doctor rather than a junior doctor (mean 4.50 vs. 2.92 on a 5‐point scale) [10]. Nonetheless, that study also found that the teacher's communication skills were rated as more important than their career stage [10]. Previous studies have shown that more experienced resident doctors receive slightly better clinical teaching ratings from students and supervised doctors than less experienced ones [6, 19].

Students had gained the most internship experience at university hospitals and in the fields of internal medicine. Accordingly, these settings were most frequently rated best for clinical teaching. Students' reasoning for choosing internal medicine doctors, included their willingness to teach and their encouragement of active student participation, and this aligns with traits identified as characteristics of effective instructors in a previous publication based on the second part of our survey [16]. Additionally, the internal medicine rotation occurs early in the clinical studies and is relatively long compared to rotations in many other specialties. This may partly explain the ranking of internal medicine.

Students who selected university hospitals often cited reasons such as the doctors' familiarity with clinical teaching and their sense of duty to teach. University hospitals, being teaching institutions, have a long‐standing commitment to developing both general medical teaching and clinical teaching to meet institutional needs [20]. Consequently, a significant portion of clinical teaching takes place in university hospitals. Therefore, students might prefer these settings due to their familiarity. Contrarily, Bennett et al. found that students rated smaller hospitals more favourably than larger ones [21]. Supporting this, an Australian study reported that students in rural programmes outperformed those in metropolitan programmes academically over the course of a year [22].

Naturally, internship experience had the most significant impact on the responses of 4th‐year students, who otherwise had very limited clinical teaching experience. Surprisingly, most 5th‐ and 6th‐year students also reported receiving the best clinical teaching in the field and place where they had gained their internship experience. Additionally, students may apply for internships in fields in which they are highly motivated. Previous studies have found a positive correlation between students' motivation in a subject and the evaluations they give [23, 24].

However, the finding that most students rated their best clinical teaching occurring in the places and fields where they had internship experience was clear. Taking this into account, the varying levels of experience in being supervised by junior and senior doctors may explain previous contradictory results in student evaluations. Additionally, experience from different training sites must be considered in future studies when evaluating students' ratings of the best clinical teaching places. Similarly, we should not conclude that clinical teaching should be exclusively conducted in the places rated best by students. Instead, the effective teaching practices identified should be implemented across various settings.

A characteristic identified for doctors who provide effective clinical teaching, as frequently highlighted in open responses, was an interest in teaching. This finding aligns with a survey conducted in Pakistan, which identified teaching interest as the most crucial characteristic for instructors [25]. It is noteworthy that nearly all aspects mentioned in the open responses were not entirely specific to particular fields or locations. Some respondents indicated that interaction is more comfortable with near‐peers. Notably, young doctors and resident doctors often belong to the same group, and students' justifications for both were very similar. Additionally, students appreciated that those groups often remembered and understood their knowledge, skill level, and specific needs. However, these qualities are not exclusive to younger doctors; more experienced doctors can also facilitate interaction by being enthusiastic about teaching. Assessing students' study phase and understanding of the subject enables effective mapping of their knowledge [26].

### 4.1 Limitations

Our study has several limitations. Firstly, all respondents are from a single faculty, and the local curriculum may have influenced their responses. As a confounding factor, it is important to consider the students' uneven exposure to different fields and settings, and the fact that most clinical rotations take place in the university hospital. These differences were also evident in internship experiences, which tended to favour internal medicine and university hospitals. Kappa values calculated from the pilot study were found to be partially low, which should be considered when interpreting the results. Additionally, the classification of specialty fields is not universal, and country‐specific differences in medical education limit the generalisability of the results outside of Finland.

On the other hand, the relatively high response rate can be considered to be a strength of our study. Furthermore, we were able to compare responses from students in different years of study, adding depth to our analysis.

## 5 Conclusion

Our study supports the notion that students most commonly prefer to learn with younger doctors and helps clarify the previously discrepant literature [9, 27]. Doctors at various career stages have been found to impact learning differently and possess distinct teaching characteristics [6, 28]. Similarly, in our study, students justified their preferences for different groups based on slightly varying reasons. Our findings suggest that organisations aiming to enhance clinical teaching should allocate sufficient time for it. In the face of growing medical demands and ever‐limited resources, it could be beneficial to allocate junior doctors to be mainly responsible for training medical students, and senior doctors to be mainly responsible for extending the experience of junior doctors. Doctors can enhance students' satisfaction with clinical teaching by demonstrating willingness to teach and by understanding the students' skill levels and learning needs. Moreover, doctors should encourage active student participation.

*Doctors can enhance students' satisfaction with clinical teaching by demonstrating willingness to teach and by understanding the students' skill levels and learning needs*.

## Author Contributions

**Verneri Hannula:** conceptualization, methodology, formal analysis, visualization, writing – original draft, writing – review and editing, data curation. **Lari Lehtovirta:** conceptualization, methodology, supervision, writing – review and editing, project administration. **Hermanni Liu:** formal analysis, writing – review and editing, data curation. **Markku Sumanen:** conceptualization, methodology, project administration, supervision, writing – review and editing.

## Conflicts of Interest

The authors declare no conflicts of interest.

## Supporting information


**Data S1:** Questionnaire.

**Data S2:** Fields of specialities.

## Data Availability

The data that support the findings of this study are available on request from the corresponding author.
