# Supplementary material for: Medical Students' Perceptions of the Best Clinical Teaching
Source: Clin Teach. 2025 Jun 13;22(4):e70119. doi: 10.1111/tct.70119 (PMC12165778; doi:10.1111/tct.70119)
Supplement: Supplementary file 1 — Data S1: Questionnaire. [file TCT-22-e70119-s001.docx]

**Medical students’ perceptions of the best clinical teaching**

The questionnaire used in the survey. Translated from the original Finnish form.

1. Your year of study?

- 4^th^ year
- 5^th^ year
- 6^th^ year

1. Your gender?

- Male
- Female
- Other

1. Your age? (Answer a number)

______________________

1. Where have you had internships? (You can select multiple answers)

- University hospital
- Central hospital
- Other hospital
- Health centre

1. In which fields have you had internships?

- Fields of surgery
- Other operative fields
- Fields of internal medicine
- Other conservative fields
- Fields of psychiatry
- Diagnostic fields
- Other fields

1. What is the total length of your internships combined? (Answer a number)

______________________

From the next question, answer the questions regarding your internship experiences, clinical practice, and health centre visits.

1. From which training-phase physician do you feel you have received the best clinical teaching?

- Specialists
- Specialising physicians
- Graduated, but not specialising physicians
- Bachelors of Medicine
- The training phase does not matter

1. Open question to the previous one, why?

______________________

1. From which age group do you feel you have received the best clinical teaching?

- 25–34
- 35–44
- 45–54
- 55–65
- Age does not matter

1. Open question to the previous one, why?

______________________

1. From which field of physicians do you feel you have received the best clinical teaching?

- Fields of surgery
- Other operative fields
- Fields of internal medicine
- Other conservative fields
- Fields of psychiatry
- Diagnostic fields
- Other fields
- The field does not matter

1. Open question to the previous one, why?

______________________

1. From where do you feel you have received the best clinical teaching?

- University hospital
- Central hospital
- Other hospital
- Health centre
- The place does not matter

1. Open question to the previous one, why?

______________________
