# Supplementary material for: Medical Students' Perceptions of the Best Clinical Teaching
Source: Clin Teach. 2025 Jun 13;22(4):e70119. doi: 10.1111/tct.70119 (PMC12165778; doi:10.1111/tct.70119)
Supplement: Supplementary file 2 — Data S2: Fields of specialities. [file TCT-22-e70119-s002.docx]

Supplement file 2: Fields of specialities in Finland by categories.

Source: https://www.erikoisalani.fi/artikkelit/kaikki-alat (in Finnish), date accessed July 15, 2024)

| Fields of surgery | Other operative fields | Fields of internal medicine | Other conservative fields | Fields of psychiatry | Diagnostic fields | Other fields |
| --- | --- | --- | --- | --- | --- | --- |
| Cardiothoracic Surgery | Anaesthesiology and Intensive Care | Cardiology | Child Neurology | Adolescent psychiatry | Clinical Chemistry | Forensic medicine |
| Gastroenterological Surgery | Emergency Medicine | Endocrinology | Dermatology and Allergology | Child psychiatry | Clinical Genetics | General practice |
| General surgery | Obstetrics and Gynaecology | Clinical Haematology | Geriatrics | Forensic psychiatry | Clinical Microbiology | Occupational health |
| Hand Surgery | Ophthalmology | Gastroenterology | Neurology | Psychiatry | Clinical Neurophysiology | Public Health |
| Neurosurgery | Otorhinolaryngology | Infectious Diseases | Oncology |  | Clinical Pharmacology and Pharmacotherapy | Sports Medicine |
| Oral and Maxillofacial Surgery | Phoniatrics | Internal Medicine | Paediatrics |  | Clinical Physiology and Nuclear Medicine |  |
| Orthopaedics and Traumatology |  | Nephrology | Physical and Rehabilitation Medicine |  | Pathology |  |
| Paediatric Surgery |  | Rheumatology | Respiratory Medicine and Allergology |  | Radiology |  |
| Plastic Surgery |  |  |  |  |  |  |
| Urology |  |  |  |  |  |  |
| Vascular Surgery |  |  |  |  |  |  |
